# Smokers show an altered hemodynamic profile to active stress: Evidence of a dysregulated stress response in young adults

**DOI:** 10.1111/psyp.14081

**Published:** 2022-05-02

**Authors:** Siobhán Howard, Tracey M. Keogh, Brian M. Hughes, Stephen Gallagher

**Affiliations:** ^1^ SASHLab, Centre for Social Issues Research, Department of Psychology, Faculty of Education and Health Sciences University of Limerick Limerick Ireland; ^2^ Health Research Institute University of Limerick Limerick Ireland; ^3^ School of Psychology National University of Ireland, Galway Galway Ireland

**Keywords:** cardiovascular reactivity, hemodynamic profile, smoking, stress

## Abstract

Tobacco smoking has been associated with lower cardiovascular reactivity to psychological stress in middle‐aged samples, but its impact on cardiovascular reactivity to stress in young adults remains unclear. The present study examined whether young healthy adults showed differing cardiovascular stress reaction profiles depending on their smoking status. Across two laboratory studies (*N* = 64 and *N* = 114), we asked participants to complete cognitive stress‐tasks while undergoing continuous hemodynamic monitoring. In both studies, there was not a statistically signification association between systolic blood pressure, diastolic blood pressure, or heart rate reactivity to stress (all *p*s > .05). However, examination of the underlying hemodynamic profile of the stress response suggested differences between non‐smokers and smokers in both studies. In Study 1, non‐smokers exhibited the expected myocardial response to the active stress‐task; however, smokers exhibited a mixed hemodynamic profile. In Study 2, smokers evidenced a weaker myocardial profile to the active stress‐tasks compared to non‐smokers. However, the examination of the continuous hemodynamic profile score (HP) did not identify statistical differences. These results highlight that any level of the smoking habit is associated with an altered hemodynamic profile in response to stress in smokers, which may have important implications for long‐term cardiovascular health. The findings also suggest that controlling for smoking behavior in reactivity research examining blood pressure and heart rate responses to stress in young adults is not necessary.

## Abstract 2

Tobacco smoking has been associated with lower cardiovascular reactivity to psychological stress in middle‐aged samples, but its impact on reactivity in young adults is unclear. In this two‐study article, we find that while smoking tobacco is not associated with blood pressure or heart rate reactivity to stress in young adults, it is associated with a mixed hemodynamic profile. This indicates that smoking is associated with a dysregulated stress response in young adults.

## 1 INTRODUCTION

The impact of tobacco smoking on cardiovascular disease risk is well‐established (World Health Organisation, 2012) and is the leading behavioral risk factor in a large number of preventable deaths worldwide. It is causally related to cardiovascular disease and is linked to atherosclerosis that starts in adolescence (e.g., Strong et al., 1992). The impact of tobacco smoking on the cardiovascular system is immediate, with both blood pressure and heart rate (HR) increasing immediately during smoking (e.g., Hasenfratz & Battig, 1992; James & Richardson, 1991). This appears to reflect the biochemical impact of nicotine on physiology (Heishman et al., 1993) rather than the physical consequences of respiratory inhalation (as effects are not seen with sham smoking; Hori et al., 1994). Despite these effects on the steady functioning of the cardiovascular system, the impact of smoking on cardiovascular *reactivity* to psychological stress is not entirely clear. This is important as the cardiovascular reactivity hypothesis posits that exaggerated or sustained cardiovascular reactions in response to psychological stress confer damage to the cardiovascular system that leads to future disease risk (Chida & Steptoe, 2010; Obrist, 1981), with blunted cardiovascular reactions to stress also predictive of future ill‐health (Phillips et al., 2013). Therefore, in addition to its direct effect on health, smoking may further contribute to long‐term disease risk by disrupting the cardiovascular response to daily stress.

In studies employing the cardiovascular reactivity paradigm, control of confounding variables when assessing the impact of psychological stress on cardiovascular reactions is important. One variable that is controlled with varying degrees of consistency across studies is smoking status. Tobacco smokers are often excluded entirely from participation in laboratory studies or are asked to restrict their nicotine intake for an arbitrary time period; alternatively, smoking status is used as a control variable in statistical analyses. The aim of such approaches is to control for the well‐established impact of smoking on the cardiovascular system.

While it appears that smoking is associated with blunted cardiovascular reactions in cross‐sectional studies (e.g., Ginty et al., 2014; Phillips et al., 2009; Sheffield et al., 1997), asking participants to refrain from smoking prior to a laboratory session does not appear to impact on cardiovascular reactions (e.g., al'Absi et al., 2003; Robinson & Cinciripini, 2006). Moreover, it appears that the chronic damage to the cardiovascular system caused by long‐term smoking in middle‐aged samples cannot be offset by short‐term restrictions on smoking prior to laboratory sessions (e.g., Sheffield et al., 1997).

Smoking has repeatedly been demonstrated to be associated with blunted cardiovascular reactivity in middle‐aged persons (e.g., Ginty et al., 2014; Phillips et al., 2009; Sheffield et al., 1997), with lower blood pressure and HR reactions to stress reported. What is less well‐established is whether smoking has a similar impact on younger populations. Both Dobkin et al. (1998) and Evans et al. (2012) have shown younger smokers to exhibit lower systolic blood pressure (SBP) and HR reactivity to stress respectively. However, among the relatively few other studies to have been conducted, a variety of intriguing patterns have been observed (e.g., Back et al., 2008; Childs & De Wit, 2009). For example, Hughes and Higgins (2010) analyzed an anthropometrically matched dataset of young adult smokers and non‐smokers and found that, while there were no differences in cardiovascular reactivity between the smokers and non‐smokers, female non‐smokers uniquely demonstrated sensitization (i.e., increases) in their diastolic blood pressure (DBP) responses across consecutive exposures to stress.

The seemingly conflicting findings between blunted cardiovascular reactivity consistently reported in middle‐aged samples of smokers but not in younger samples may be explained by examination of the underlying hemodynamic profile of the stress response. Blood pressure is a cumulative outcome of underlying processes of blood flow and vascular resistance. As such, any given measure of blood pressure change will reflect a combination of a person's cardiac output (CO) and total peripheral resistance (TPR). Changes in blood pressure, such as those that occur in response to stress, will be a function of changes in CO and TPR, either singly or in combination (Turner et al., 1994). While examination of cardiovascular reactions to stressful stimuli has traditionally focused on the pressor responses of SBP, DBP, and HR, a more in‐depth examination of the profile of the cardiovascular reaction is possible, allowing researchers to characterize different stress responses by type. Differing hemodynamic patterns underpinning stress reactivity were reported as far back as the 1980s (e.g., Light et al., 1993; Light & Sherwood, 1989; Manuck et al., 1990). With advances in technology, it has since become significantly easier to measure CO and TPR in laboratory studies. As such, the inclusion of such hemodynamic variables and the examination of hemodynamic patterning may improve upon the modest associations reported between stress reactivity and disease risk in the past (e.g., Kamarck & Lovallo, 2003; Ring et al., 2002).

Gregg et al. (2002) developed a computational model to represent individual differences in such hemodynamic patterning, focussing on the reciprocal relationship between CO and TPR as biomechanical variables. This so‐called hemodynamic profile/compensation deficit model (HP/CD model) of blood pressure regulation has been applied across a number of subsequent studies (see James et al., 2012; Ottaviani et al., 2017). The model identifies HP as a measure of cardiovascular response type (ranging on a continuum from *vascular* to *cardiac*, with *mixed* as a mid‐point) and CD as a measure of the magnitude of the response exhibited. The model provides for the computation of quantitative scores for both HP and CD should they be necessary for tests of statistical significance; one‐sample *t* tests indicate if a HP score is significantly above zero (indicating a vascular response) or below zero (indicating a myocardial response); scores not differing from zero indicating a mixed hemodynamic response to the stressor. The inclusion of HP in reactivity studies allows researchers to characterize the stress response profile as myocardial (changes in blood pressure are driven by CO), vascular (changes in blood pressure are driven by increases in TPR), or mixed (changes are driven by increases on both parameters).

The HP/CD model has been used to show that a vascular or mixed HP during stress is associated with several known cardiovascular risk factors, such as elevated ambulatory pulse pressure (Gregg et al., 2005), Type D personality (Howard et al., 2011), sleep deprivation in healthy young adults (James & Gregg, 2004; O'Leary et al., 2013), caffeine intake (James & Gregg, 2004), and perseverative cognition (Ottaviani et al., 2017). Further, in many of these studies, a so‐called mixed HP appears to underpin what in other studies is identified as blunted cardiovascular reactivity (e.g., Howard et al., 2011), and crucially, to differentiate between groups that would otherwise be measured as having equivalent blood pressure responses to stress (e.g., James & Gregg, 2004; Ottaviani et al., 2017). In other words, while changes in such parameters as SBP, DBP, or HR responses might appear consistent across different groups, patterns of HP might well reveal that some of these responses are driven by vascular factors, while other responses are driven by cardiac (or even mixed) ones. It may be that differences in cardiovascular stress responses between middle‐aged smokers and young adult smokers show consistency in their hemodynamic response profiles; blunted HR reactivity in particular is likely to be underpinned by a more vascular pattern of response.

Active stress tasks usually elicit a more myocardial HP, dominated by changes in CO. It is generally accepted that if active stress elicits a mixed or vascular HP, with greater impact from changes in TPR, then this is potentially more damaging to the cardiovascular system and contributes to disease risk (Gregg et al., 2002). People who typically respond with increased TPR that is not accompanied by a compensatory decrease in CO (i.e., vascular reactors) are at increased disease risk due to vasoconstriction and subsequent vascular changes (Obrist, 1982). Similarly, people who exhibit a mixed HP (i.e., mixed reactors) are liable to experience hypertension, reflecting a cumulative abnormality in the CO‐TPR homeostasis (Hejl, 1957). As such, examination of these different patterns of reactivity could have important implications for understanding cardiovascular disease and may be particularly revealing when examining conflicting findings in relation to the pressor responses of SBP and DBP. This may also help elucidate some of the underlying mechanisms linking smoking, stress, and heart disease (Epstein & Perkins, 1988).

Consequently, the aim of the present study was to examine if the underlying hemodynamic profile of the stress reaction in young adults varied depending on their smoking status. Two studies were conducted to examine if any degree of smoking habit had an effect on the hemodynamic profile of the stress response. Based on previous research, it was anticipated that there would be little differences in SBP, DBP, and HR reactivity between young adult smokers and non‐smokers. However, given that smoking is associated with increased cardiovascular disease risk, as well as blunted reactivity in middle‐aged samples, it was hypothesized that young smokers would show a mixed or vascular hemodynamic profile to the active stress‐tasks.

## 2 STUDY 1

### 2.1 Method

#### 2.1.1 Design

Study 1 employed a 2 × 2 mixed factorial design. The within‐subjects factor was the phase with two levels; baseline and task. The between‐subjects factor was smoking status; smoker or non‐smoker. The dependent variables were SBP, DBP, HR, CO, and TPR. The reaction profile was characterized using the HP/CD model, yielding an additional dependent variable of HP. Computational details of this model are provided below.

#### 2.1.2 Participants

Participants were drawn from a sample of 84 college students who underwent cardiovascular monitoring throughout a traditional laboratory protocol. Inclusion criteria were; tested as normotensive in the laboratory (SBP < 140 mmHg and DBP < 90 mmHg) and age < 30 years, to ensure a young‐adult sample. An overview of the number of participants excluded due to each criteria can be seen in Figure S1. This left a sample size of 66 college students, ranging in age from 18 years to 27 years (*M* = 19.54, *SD* = 1.72). There were 22 self‐identified smokers (13 of whom identified themselves as social smokers) and 44 non‐smokers. One individual identified themselves as an ex‐smoker and a second identified themselves as an e‐cigarette smoker; these individuals were removed from the analyses, leaving a sample of 64 participants (49 women, 15 men), ranging in age from 18 to 21 years (*M* = 19.47, *SD* = 1.64), with mean body mass index (BMI) of 23.28 kg/m^2^ (*SD* = 3.62). There were 21 smokers and 43 non‐smokers. Thirty‐one of the women identified that they were taking hormonal contraceptives, 8 of whom were smokers. Ethical approval was obtained from the institutional research ethics committee.

Post‐hoc power analysis by G Power (Faul et al., 2009) for a one‐sample *t* test, showed 89% power to detect a medium effect in the sample of 43 and just 58% power to detect a medium effect in the subsample of 21. There was 50% power to detect the between‐within interaction in the sample of 64, with two repeated measures.

#### 2.1.3 Materials

##### Laboratory stress

Both the paced auditory serial addition test (PASAT) and a speech task were used to successfully elicit a cardiovascular and psychological stress response, as in previous research (e.g., Mathias et al., 2004; McMahon et al., 2021). For the PASAT, a series of single numbers from 1 to 9 were presented on a computer screen. Participants were required to add a sequence of number pairs while retaining in memory the second number for addition to the next number presented. Participants returned their answers verbally to the researcher. Four separate series were presented, each lasting 1 min with a 5‐s interval between presentations, getting progressively shorter each time. The presentation rate was 2.4, 2.0, 1.6, and 1.2 s.

For the speech task, participants were advised that they would have 2 min to prepare a speech in which they had to name and talk about three of their best and three of their worst qualities. They were then instructed that they would have 4 min to present their speech to the experimenter. The order of task presentation was counterbalanced across the study. All participants received the same instructions in advance of the tasks and the entire stress phase lasted 10 min.

##### Cardiovascular reactivity assessment

Beat‐to‐beat blood pressure and heart rate were measured non‐invasively using a Finometer hemodynamic cardiovascular monitor (Finapres Medical Systems BV, BT Arnhem, The Netherlands). The Finometer is based on the volume‐clamp method first developed by Peňaz (1973). An appropriate‐sized finger cuff is attached to the participant's middle finger which inflates to keep the arterial walls at a set diameter. In‐built into this finger cuff is an infrared photo‐plethysmograph that detects changes in the diameter of the arterial wall. When the volume clamp is active at the proper unloaded diameter, intra‐arterial pressure equals that of the finger cuff pressure. Measures of arterial pressure CO are provided based on the previously validated Modelflow modeling method (Wesseling et al., 1993, 1995). The Finometer has been shown to accurately assess absolute blood pressure in young participants (Schutte et al., 2003) and cardiac patients (Guelen et al., 2003). According to these studies, the validation criteria of the Association for the Advancement of Medical Instrumentation and the revised protocol of the British Hypertension Society are satisfied by the Finometer.

##### Self‐report questionnaires

A demographic questionnaire was used to assess self‐reports of smoking status, and other potential confounding variables, such as age, caffeine intake, physical exercise, and other sociodemographic questions. Participants were asked to indicate whether they were a current smoker, ex‐smoker, social smoker, or e‐cigarette user. Participants were also asked about the number of cigarettes they smoked per day. In addition, 10‐point Likert scales were used as manipulation checks to confirm that the participants experienced the tasks as stressful and difficult.

##### Nicotine dependence measure

The Brief Wisconsin Inventory of Smoking Dependence (Brief WISDM) questionnaire (Smith et al., 2010) was used to assess nicotine dependence. This 37‐item questionnaire consists of 11 subscales and is a multidimensional measure of nicotine dependence. Each item is answered on a 7‐point Likert scale ranging from (1) “not true of me at all” to (7) “extremely true of me”. Smith et al. report good psychometric properties across three independent samples. In the present sample, Cronbach's *α* of .96 for the 37‐item scale confirmed that this questionnaire had excellent internal consistency.

#### 2.1.4 Procedure

Participants were asked to avoid exercise and alcohol consumption for 12 hr prior to their participation, along with caffeine and nicotine for 2 hr. This was confirmed by self‐report on the participant's arrival at the laboratory. On arrival, participants were seated at a computer desk in a comfortable chair with an arm support. A personal computer was situated on the desk. The Finometer cuff was attached to the participant's middle finger of their non‐dominant hand. Participants were given 20 min to acclimatize to the laboratory situation during which the self‐report questionnaires were completed, including ratings of how difficult and stressful they expected the tasks to be. Reading material was also supplied in order to facilitate relaxation and the establishment of cardiovascular baselines, by offsetting the risk of rumination‐related arousal (Jennings et al., 1992). After the initial 20‐min acclimatization period, participants were instructed to relax quietly for 10 min. Resting measures were obtained during this time. Following this resting baseline period, participants were asked to perform the PASAT and the speech task. Immediately before the stress task began, the main laboratory light was turned off, leaving only light from a desk lamp. Participants were instructed by the researcher, who wore a white laboratory coat, to give the answers to the arithmetic task orally. There were no other evaluative components to the task. At the end of the task period, participants completed the self‐report scales.

#### 2.1.5 Overview of analyses

Independent *t* tests and Mann Whitney were used to ensure both groups were comparable on potential confounding variables. Chi‐squared tests of association were used where the variables were of a nominal scale of measurement. Mixed‐factorial ANOVAs were used to test the main and interaction effects of the variables on performance and experience during the task, as well as on the cardiovascular parameters. Independent *t* test was used to examine if HP differed between smokers and non‐smokers while one‐sample *t* tests were used to identify if the nature of the stress reaction was altered between smokers and non‐smokers.

Effect sizes are presented as partial *η* ^2^ for ANOVA effects. Partial *η* ^2^, rather than simple *η* ^2^, is recommended for ANOVA designs with multiple independent variables, as simple *η* ^2^ contains systematic variance attributable to other effects and interactions (Tabachnick & Fidell, 1989). Eta‐squared values of .01, .09, and .25 are taken as representing small, medium, and large effect sizes, respectively (Cohen, 1988, 1992).

### 2.2 Results

#### 2.2.1 Data reduction

Mean levels of all cardiovascular parameters were computed for the baseline and the combined task phases of the protocol. A mean task value in relation to the combined PASAT and speech task was computed and this was taken as the task‐level mean. Based on the baseline and the mean of the two tasks, HP was computed using trigonometric rotation (Gregg et al., 2002; James et al., 2012). The computation is based on the following equation:

$$\log\left(\mathrm{CO}\right)r+\log\left(\mathrm{TPR}\right)r=\log\left(\text{mean arterial pressure}\right)r$$

where r indicates a ratio of the task to baseline values. Higher values of HP indicate a vascular profile of reactivity, as the algebraic increase in log(TPR)r exceeds that in log(CO)r. The formula used to calculate HP was as follows;

$$\left(\left(\mathrm{Lg}10\left[\mathrm{TPR}\_\text{Task}/\mathrm{TPR}\_\text{Baseline}\right]\right)–\left(\mathrm{Lg}10\left[\mathrm{CO}\_\text{Task}/\mathrm{CO}\_\text{Baseline}\right]\right)\right)/\text{SQRT}\left(2\right)$$

#### 2.2.2 Potential confounding variables

Independent samples *t*‐tests confirmed that there were no differences between the two groups in age or BMI. Likewise, there were no differences in the number of caffeinated products consumed in a week, exercise taken, or alcohol consumed (all *p*s > .120).

#### 2.2.3 Manipulation check

Of the 22 smokers, 7 identified as being current smokers and 13 as social smokers. Independent *t*‐test confirmed that current smokers had higher nicotine dependence (*M* = 36.77, *SD* = 7.48) compared to social smokers (*M* = 17.74, *SD* = 5.92), *t*(17) = 6.15, *p* < .001, [95% CI, 12.50, 25.57]. Smokers smoked on average 5.00 cigarettes a day (*SD* = 1.63), whereas social smokers smoked a mean of 1.12 cigarettes a day (*SD* = 1.42)*, t*(18) = 5.55, *p* < .001, [mean difference = 3.89, 95% CI, 2.42, 5.35]. Smokers were smoking for an mean of 3.71 years (*SD* = 3.35), whereas social smokers reported smoking for a mean of 1.23 years (*SD* = 1.36), *t*(7.09) = 1.88, *p* = .102.

2 × 2 mixed ANOVAs confirmed that participants expected the PASAT task to be more stressful than the speech task, *F*(1, 61) = 30.40, *p* < .001, partial *η* ^2^ = .33, and more difficult, *F*(1, 61) = 51.93, *p* < .001, partial *η* ^2^ = .40; smoking did not moderate this difference (both *p*s > .173. After the task, participants reported that they found the PASAT more stressful, *F*(1, 62) = 19.66, *p* = .001, partial *η* ^2^ = .24, and more difficult, than the speech task, *F*(1, 62) = 19.66, *p* = .001, partial *η* ^2^ = .24. As with expectations, smoking status did not moderate this difference (both *p*s > .249). Participants found the PASAT more stressful than they expected, *F*(1, 61) = 17.86, *p* < .001, partial *η* ^2^ = .23, as well as more difficult, *F*(1, 61) = 35.47, *p* < .001, partial *η* ^2^ = .37. Likewise, participants found the speech task more stressful, *F*(1, 61) = 11.37, *p* = .001, partial *η* ^2^ = .216, and more difficult than they expected, *F*(1, 61) = 18.47, *p* < .001, partial *η* ^2^ = .23. Smoking status did not moderate these effects (all *p*s > .123). Interestingly there was a main effect for smoking status on ratings of stress and difficulty with respect to the speech task, where non‐smokers returned higher stress ratings, *F*(1, 61) = 4.28, *p* = .043, partial *η* ^2^ = .07, and difficulty ratings, *F*(1, 61) = 4.12, *p* = .047, partial *η* ^2^ = .06. Independent samples *t* tests confirmed that there were no differences on PASAT score between smokers and non‐smokers, *t*(161) = .59, *p* = .555.

Main effects for phase on all cardiovascular measures confirmed that the task was successful in eliciting reactivity; SBP, *F*(1, 62) = 172.59, *p* < .001, partial *η* ^2^ = .74, DBP, *F*(1, 62) = 170.74, *p* < .001, partial *η* ^2^ = .73, HR, *F*(1, 62) = 52.54, *p* < .001, partial *η* ^2^ = .66, CO, *F*(1, 62) = 41.42, *p* < .001, partial *η* ^2^ = .40, and TPR, *F*(1, 62) = 5.42, *p* = .023, partial *η* ^2^ = .08. As can be seen in Table 1, cardiovascular parameters were higher during task compared to baseline, with the exception of TPR which was lower during the task phase.

**TABLE 1:** Mean (with *SD*) levels of cardiovascular parameters during the procedure by smoking status (Study 1)

	Smoking status			
	Smoker (*n* = 21)		Non‐Smoker (*n* = 43)	
	Mean	*SD*	Mean	*SD*
*Baseline*				
SBP (mmHg)	122.32	9.61	122.67	9.50
DBP (mmHg)	71.97	5.64	72.78	6.68
HR (bpm)	81.34	12.48	79.62	11.72
CO (lpm)	6.65	1.63	5.54	1.08
TPR (pru)	.87	.21	1.06	.21
*Task*				
SBP (mmHg)	140.33	13.97	142.88	13.53
DBP (mmHg)	83.44	8.61	86.18	10.35
HR (bpm)	87.41	14.03	88.85	12.58
CO (lpm)	7.43	1.82	6.40	1.37
TPR (pru)	.93	.27	1.12	.32

#### 2.2.4 Smoking and cardiovascular reactivity

There were no main effects for smoking on mean SBP, *F*(1, 62) = 59.65, *p* = .603, DBP, *F*(1, 62) = .79, *p* = .375, or HR, *F*(1, 62) = .002, *p* = .964, across the procedure. However, there were main effects for smoking on CO, *F*(1, 62) = 19.16, *p* = 004, partial *η* ^2^ = .13 and TPR, *F*(1, 62) = 8.15, *p* = .006, partial *η* ^2^ = .12. As can be seen in Table 1, smokers had higher CO over the procedure but lower TPR. There were no differences in SBP, DBP, or HR.

There were no phase × smoking interaction effects for SBP, *F*(1, 62) = .58, *p* = .451, DBP, *F*(1, 62) = 1.03, *p* = .314, HR, *F*(1, 62) = 2.24, *p* = .140, CO, *F*(1, 62) = .08, *p* = .779, or TPR, *F*(1, 62) = .00, *p* = .997. This confirmed that reactivity to the task was similar for smokers and non‐smokers.

#### 2.2.5 Smoking and hemodynamic profile

**FIGURE 1:**
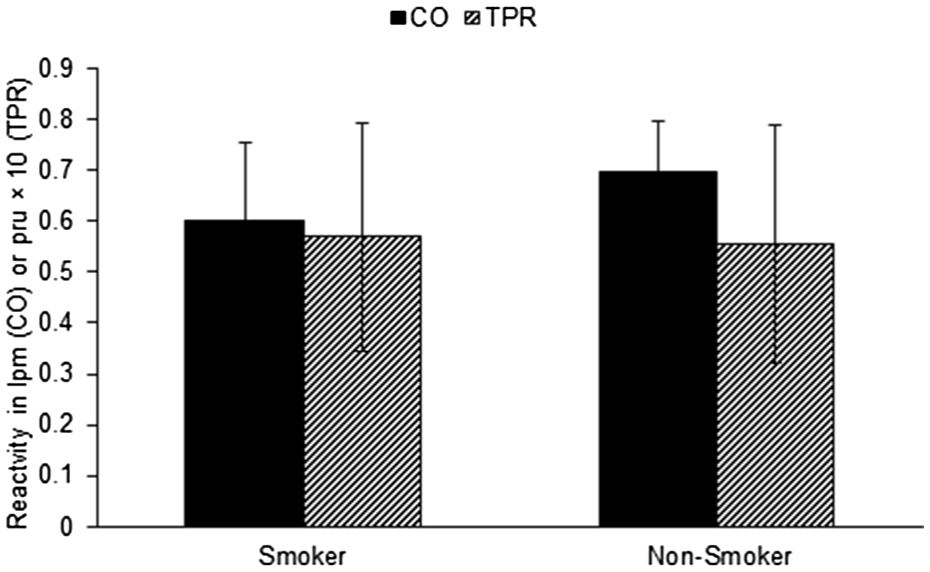
Smoking status is associated with a mixed hemodynamic response to stress. CO reactivity is in liters per minute. TPR reactivity is in peripheral resistance units

Although CO and TPR reactivity in isolation were not moderated by smoking status, to fully scrutinize the hemodynamic profile of the stress reaction, HP was computed. While an independent *t‐*test confirmed there were no differences on HP scores between smokers and non‐smokers, *t*(62) = .64, *p* = .524, [mean difference = .0146, 95% CI, −.0310, .0603], Cohen's d = .14, one‐sample *t* tests conducted on each group separately confirmed a significant difference from zero for HP for non‐smokers, *t*(42) = −2.69, *p* = .010, [mean difference = −.0318, 95% CI, −.0557, −.0079], but not for smokers, *t*(20) = −.77, *p* = .446, [mean difference = −.0172, 95% CI, −.0632, .0289]. The negative *t*‐value indicates that the reaction to the active stressor in non‐smokers was myocardial, as expected from an active task. However, for smokers, HP did not differ from zero indicating that the profile was mixed. As can be seen in Figure 1, however, both smokers and non‐smokers showed elevations in both CO and TPR, although TPR reactivity was lower in non‐smokers.

#### 2.2.6 Summary

Study 1 identified that participants who had a current smoking habit had a mixed hemodynamic profile in response to the combined active stress tasks in this study, while participants who had never engaged in a smoking habit had the expected myocardial response profile. This was despite equivalent blood pressure and HR responses to the task. Overall, however, smokers had higher CO and lower TPR across the procedure.

## 3 Study 2

### 3.1 Introduction

To test if the findings arising from Study 1 could be replicated in a similar but different sample of participants, a larger second dataset was scrutinized. The second dataset allowed us to examine whether the effects observed in Study 1 for smokers would apply to people who were currently non‐smokers but had smoked in the past. If smoking is indeed associated with altered hemodynamic profile stress response, then, even in a young adult sample, smokers, whether current or ex‐smokers, should show altered patterns of HP compared to non‐smokers, perhaps indicating chronic damage to the cardiovascular system.

### 3.2 Method

#### 3.2.1 Design

This study employed a 2 × 2 mixed factorial design. The within‐subjects factor was the phase with two levels; baseline and task. The between‐subjects factor was smoking status; previous or current smoker and non‐smoker; for ease of communication, these groups are referred to as smokers or non‐smokers. The dependent variables were SBP, DBP, HR, CO, and TPR. As in Study 1, the reaction profile was characterized in terms of the HP/CD model.

#### 3.2.2 Participants

Participants were drawn from a large sample of 134 college students who had undergone a traditional laboratory stress‐testing session. Of these 134 students, 115 (26 male, 89 female) met the inclusion criteria of; being tested as normotensive (SBP ≤ 140 mmHg and DBP ≤ 90 mmHg) and aged less than 30 years. One participant did not respond to the question regarding past smoking habits, although they did indicate they were a non‐smoker; however, given the creation of the groups to reflect on never having engaged in a smoking habit or not, this individual was removed from the analyses, leaving a sample of 114 participants (88 female, 26 male) ranging in age from 18 to 29 years (*M* = 20.14, *SD* = .46). These participants formed part of the smaller sample in a previously published study examining Type D personality and stress reactivity in women (Howard et al., 2011). The sample comprised 67 participants who had never smoked (non‐smokers) and 47 participants who either engaged in a current smoking habit (*n* = 24) or had a past smoking habit (*n* = 23). Ex‐smokers had given up smoking for a mean of 23.52 months (*SD* = 27.72), although this ranged from just 1 month to 96 months (8 years). Eleven women reported using oral contraceptive pills, nine of whom were also smokers. Current smokers reported smoking a mean of 7.52 cigarettes a day (*SD* = 5.18). Mean BMI was 22.88 kg/m^2^ (*SD* = 3.41). Chi‐squared test of association confirmed there was no association between sex and smoking status, *χ* ^2^ = 4.64, *p* = .098, while independent samples *t* test confirmed that smokers and non‐smokers had equivalent BMI, *t*(112) = .62, *p* = .539; smokers (*M* = 21.00, *SD* = 3.18) were slightly older than non‐smokers (*M* = 19.54, *SD* = 1.56), *t*(112) = 3.26, *p* = .001, [95% CI, .57, 2.35]. Ethical approval had been obtained from the institutional research ethics committee.

Post hoc power analysis by G Power (Faul et al., 2009) for one‐sample *t*‐test, showed 91% power to detect a medium effect in the sample of 47% and 98% power to detect a medium effect in the subsample of 67. There was 75% power to detect the between‐within reaction in the sample of 114, with two repeated measures.

#### 3.2.3 Materials

##### Laboratory stress

In this study, a computerized mental arithmetic task had been used to elicit cardiovascular responses to psychological stress. Subtraction problems appeared on‐screen and participants were required to solve these problems. Answers were inputted using a computer keypad. The level of difficulty varied according to the answers given, controlling for mathematical ability and employing the principle of standardized flexibility previously recommended for CVR assessment (Hughes, 2001; Turner, 1994; Turner et al., 1986). The task was time‐pressured: participants were given 15 s to return each solution; otherwise, their response was coded as a “timeout” and the next item was shown.

##### Cardiovascular assessment and self‐report questionnaires

As in Study 1, a Finometer was used to measure cardiovascular reactivity to the stress‐tasks, and a demographic questionnaire was used to assess self‐reports of smoking status, age, and other sociodemographic variables. Participants were asked to indicate if they were a current smoker or not; and if not, had they smoked in the past. If they indicated they were an ex‐smoker, they were asked to indicate how long ago had they quit. Smokers were asked about the number of cigarettes they smoked per day. In addition, 10‐point Likert scales were used as manipulation checks to confirm that the participants experienced the tasks as stressful. Participants rated how stressful, difficult, and enjoyable they found the task.

#### 3.2.4 Procedure

The overall procedure employed in Study 2 was similar to that of Study 1. In brief, participants were given 30 min to acclimatize to the laboratory environment during which they completed the demographic questionnaire and were provided with reading material. A 10‐min formal baseline followed. After the baseline, participants completed the 5‐min mental arithmetic task and the self‐reported rating scales. During the procedure, the researcher was separated from the participant by an opaque screen to limit any social‐evaluative element to this stress‐task.

#### 3.2.5 Overview of analyses

Paired sample *t* tests and ANOVA were used to confirm that the tasks were experienced as stressful. A series of 2 × 2 mixed‐factorial ANOVAs was conducted to establish that the task was successful in eliciting cardiovascular reactivity, as well as to identify if smoking status was associated with altered patterns of reactivity to the task. Independent‐samples *t* test was used to compare the HP between the groups, while one‐sample *t* tests were used to identify if HP differed from zero, in order to characterize the hemodynamic profile of the stress response. As with Study 1, mean levels of cardiovascular parameters were calculated for the baseline phase and the task phase of the procedure.

### 3.3 Results

#### 3.3.1 Manipulation checks

Paired sample *t* tests confirmed that participants rated the task as more stressful and difficult than enjoyable (all *p*s ≤ .001). Independent samples *t* tests confirmed that smokers and ex‐smokers found the task equally stressful, difficult, and enjoyable (all *p*s > .718). Independent *t* tests also confirmed that there were no differences in the number of correct or incorrect answers to the mental arithmetic task, or time‐outs, between smokers and non‐smokers (all *p*s > .178), confirming that all the participants were equally engaged in the task.

Main effects for phase on all cardiovascular measures, except TPR, confirmed that the task was successful in eliciting reactivity; SBP, *F*(1, 112) = 162.98, *p* < .001, partial *η* ^2^ = .59, DBP, *F*(1, 112) = 183.06, *p* < .001, partial *η* ^2^ = .62, HR, *F*(1, 112) = 1.68, *p* < .001, partial *η* ^2^ = .45, and CO, *F*(1, 112) = 126.89, *p* < .001, partial *η* ^2^ = .53. There was no main effect for phase on TPR, *F*(1, 112) = .79, *p* = .376. As can be seen in Table 2, levels were higher during task compared to baseline.

**TABLE 2:** Mean (with *SD*) levels of cardiovascular parameters during the procedure by smoking status (Study 2)

	Smoking status			
	Smoker (*n* = 47)		Non‐smoker (*n* = 67)	
	Mean	*SD*	Mean	*SD*
*Baseline*				
SBP (mmHg)	118.61	15.08	120.61	12.50
DBP (mmHg)	70.23	8.63	71.21	8.43
HR (bpm)	81.03	10.20	80.21	10.24
CO (lpm)	6.25	1.15	6.11	1.27
TPR (pru)	0.97	0.39	0.95	0.24
*Task*				
SBP (mmHg)	129.78	16.94	130.17	14.34
DBP (mmHg)	76.78	9.76	77.56	9.15
HR (bpm)	86.61	11.50	87.53	13.17
CO (lpm)	7.09	1.50	7.05	1.71
TPR (pru)	0.97	0.47	0.91	0.24

#### 3.3.2 Smoking and cardiovascular reactivity

There were no main effects for smoking on mean cardiovascular levels across the procedure; SBP, *F*(1, 112) = .20, *p* = .654, DBP, *F*(1, 112) = .28, *p* = .595, HR, *F*(1, 112) = .001, *p* = .981, CO, *F*(1, 112) = .12, *p* =. 734, or TPR, *F*(1, 112) = .56, *p* = .457, indicating that regardless of smoking status, participants showed similar levels of SBP, DBP, HR, CO, and TPR.

Likewise, there were no phase × smoking interaction effects for SBP, *F*(1, 112) = .98, *p* = .325, DBP, *F*(1, 112) = .04, *p* = .836 HR, *F*(1, 112) = .1.68, *p* = .198, CO, *F*(1, 112) = .35, *p* = .553, or TPR, *F*(1, 112) = .570, *p* = .404. This confirmed that reactivity to the task was similar for smokers and non‐smokers.

#### 3.3.3 Smoking and hemodynamic profile

**FIGURE 2:**
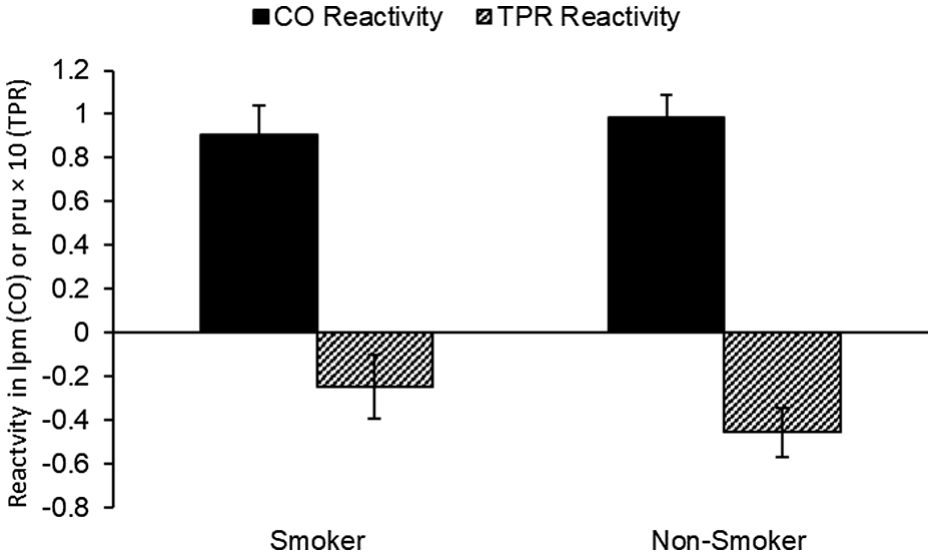
Smoking status is associated with a weaker myocardial response to stress. CO reactivity is in liters per minute. TPR reactivity is in peripheral resistance units

Independent *t* tests identified no difference on HP scores between smokers and non‐smokers, *t*(112) = .82, *p* = .42, [mean difference = .0124, 95% CI, −.0176, .0424], Cohen's d = .15. However, one‐sample *t*‐tests confirmed differences from zero for smokers, *t*(46) = −2.93, *p* = .005, [mean difference = −.0429, 95% CI, −.0724, −.0134], and non‐smokers, *t*(66) = −7.45, *p* < .001, [mean difference = −.0553, 95% CI, −.0701, −.0405] on HP identifying that for both groups, stress reaction profiles were myocardial. As can be seen in Figure 2, non‐smokers showed the healthiest myocardial response, with the compensatory increase in CO accompanied by a decrease in TPR, whereas for smokers, equivalent increases on CO to non‐smokers are evident, but are accompanied by a smaller change in TPR.

#### 3.3.4 Smokers, ex‐smokers, non‐smokers

Analyses were repeated, with smoking as a three‐level between‐subjects factor; smokers (*n* = 24), ex‐smokers (*n* = 23), and non‐smokers (*n* = 67). All findings reported above were unaltered; one‐sample *t*‐tests confirmed that the reaction profile was myocardial for all three groups, with non‐smokers showing the strongest myocardial reaction profile.

#### 3.3.5 Summary

Study 2 confirmed the findings from Study 1 that any level of smoking habit was associated with a less‐healthy hemodynamic profile in response to active stress. In Study 2, regardless of whether participants engaged in a smoking habit or not, either currently or previously, blood pressure and HR reactivity to stress were equivalent. While examination of the hemodynamic profile indicated that both groups showed clear myocardial reactions to the task it was clear that the myocardial reaction was weaker in smokers compared to non‐smokers, showing a replicable effect that smoking is associated with an altered hemodynamic profile in response to active stress.

## 4 DISCUSSION

Our results show that, in young adults, tobacco smoking is associated with disrupted cardiovascular stress responding revealed on examination of the hemodynamic profile of the stress response. The stress responses of young adults who either engage or have engaged in a smoking habit are characterized by a disrupted myocardial stress response profile to active stress. For participants who are currently engaged in any level of smoking habit (those in Study 1), a so‐called mixed HP is evident; a pattern of blood pressure change driven by non‐reciprocating shifts in both vascular and cardiac variables. Even for those who have previously engaged in a smoking habit, a disruption to the expected myocardial response is evident, as shown in Study 2. As healthy blood pressure requires TPR and CO to offset each other, so that the vascular system is protected from being overly impacted by sudden increases in blood flow (Hejl, 1957), this suggests that young smokers exhibit a type of stress response that is potentially damaging to the cardiovascular system (James et al., 2012).

This is important as it shows that even in young adults, where the chronic negative impact of smoking on the cardiovascular system may not be considered significant, we see differences emerging at this early stage in terms of stress reactivity. In addition, not only were these differences observed in young adults, they were also evident regardless of whether people reported that they were current daily smokers, social smokers, or, ex‐smokers. Further, the present analyses show that young smokers evidence an altered hemodynamic profile of response compared to non‐smokers even though their superficial blood pressure responses (in terms of SBP and DBP) are similar.

These findings, on the surface, appear to contrast with those reported by Evans et al. (2012) who showed that smoking was associated with blunted HR reactivity in response to psychological stress in a sample of adolescents. In the present study, we did not find any differences between smokers and non‐smokers in blood pressure or HR reactivity. However, the mixed and weaker myocardial profile identified in our two studies is in the same direction as that reported by Evans et al., with lower HR reactivity more likely to be reflected by a vascular profile of response, or certainly a weaker myocardial response (given that CO is a product of HR by stroke volume) rather than a myocardial profile of response that would be driven mainly by increases on CO.

Smoking is often regarded as a confounding variable in laboratory studies, with varying degrees of control exerted to account for its purported effects on blood pressure and HR. Some studies choose to recruit only non‐smokers; others restrict the intake of tobacco for an arbitrary time prior to the laboratory study. Many more give no information on the number of smokers in the sample. Indeed, in the two studies reported in this article, control of the acute effects of smoking was exerted by asking participants not to smoke for two hours prior to the laboratory session. This is done in order to ensure that the acute effects of smoking do not unduly impact on cardiovascular reactivity to stress, but also to ensure that blood pressure and HR are not impacted by any withdrawal effects if a longer restriction period was implemented. However, the few experimental studies that manipulate smoking prior or during laboratory studies have shown little effects of smoking on cardiovascular reactivity to stress, although resting and overall measures do appear to be impacted (e.g., Hasenfratz & Battig, 1991). In parallel, cross‐sectional studies have now established that smokers show smaller SBP and DBP reactions than non‐smokers. This has been reported in samples with a mean age of 41 years (Phillips et al., 2009), 58 years (Ginty et al., 2014), and in samples where the age ranged from 35–55 years (Sheffield et al., 1997). While the consensus is that the acute effects of smoking restriction cannot offset the damage done by chronic smoking status in these middle‐aged samples, the present study suggests that even in young adults, smoking is associated with altered patterns of stress reactivity when the underlying hemodynamic profile of the stress reaction is examined. It also does so in a way that is consistent with the blunted HR reactions seen later in life; a vascular profile of reactivity is more likely to be associated with blunted HR reactions given that CO is the product of HR by stroke volume. This suggests that damage to the system occurs early and identifies that smoking influences the development of cardiovascular disease, not just from its impact on the system directly, but also through its impact on the stress response. However, the findings also suggest that in young adult samples where blood pressure and HR reactions are the outcomes of interest, the smoking status of the participant is not pertinent information to either collect or control for.

The findings are also noteworthy as smoking has been associated with blunted reactions to stress, not only on HR reactivity but also on SBP and DBP reactivity. Like this study, findings have been reported when comparing any degree of smoking behavior to never smoked (Ginty et al., 2014; Phillips et al., 2009). While it may be expected that blunted HR reactions would be underpinned by a vascular or mixed hemodynamic profile, it is not the case that blunted SBP or indeed DBP reactions would necessarily follow with a vascular or mixed hemodynamic profile. While CO and HR are highly correlated, thereby a reduction in HR reactivity will often be associated with a reduction in CO change in response to stress (thereby arising as a non‐myocardial response in the HP/CD model), this is not the case for either SBP and DBP.

While it is well‐established that smoking cessation reduces the risk of coronary heart disease (e.g., Kannel et al., 1984), it appears that it takes several decades of continuous abstinence to allow the reduction of risk to approach that of those who never smoked (Ding et al., 2019; Shields & Wilkins, 2013). Certainly, in the present study, even in a young adult sample, we see that damaging patterns of stress reactivity are still exhibited even in young ex‐smokers, suggesting that the damage can never be wholly reversed, even when the individual has only smoked for a small number of years. It is also not clear from this study whether the altered patterns of stress reactivity are due to the effects of smoking on the vasculature directly, or whether smoking influences stress reactivity independently. In fact, a review of the literature in both human and animal studies linking stress, smoking, and negative affectivity failed to identify clear directions of effect, with findings showing that nicotine yields inconsistent effects on stress and negative affectivity (Kassel et al., 2003).

The HP/CD model of the hemodynamic profile has a number of strengths when examining important differences in stress reactivity that may have implications for future ill‐health. First, the model has suggested there are small alterations in the hemodynamic profile of the stress response in young adults depending on their smoking status; any degree of smoking habit was associated with a mixed or weaker myocardial response. While significant differences did not arise on examination of the HP score itself, scrutiny of the hemodynamic profile showed alterations in the stress response. This may be important in terms of future disease risk, as this pattern of findings emerged even in young adults where damage to the cardiovascular system as a result of smoking may not be apparent on examination of resting blood pressure levels or reactivity. However, it also offers some utility for future research investigating blunted reactions to stress. Lower CVR to stress has now been associated with a number of moderating or outcome variables, including obesity (Carroll et al., 2008), depression (Phillips, 2011), anxiety (Yuenyongchaiwat & Sheffield, 2017), smoking (e.g., Ginty et al., 2014; Phillips et al., 2009; Sheffield et al., 1997), as well as personality traits such as Type D (Howard et al., 2011). Such blunted blood pressure reactions may be underpinned by differing hemodynamic response profiles, which may further elucidate the health‐damaging consequences or precursors of blunted reactivity.

The HP/CD model, however, uses the computed HP variable as a way to describe the nature of the hemodynamic profile exhibited by different groups or different conditions. As such, the majority of the previous research examines whether the HP indicates a myocardial, vascular, or mixed response by testing whether HP differs significantly from zero (indicating myocardial or vascular if significantly below or above zero) or not (indicating a mixed response) (e.g., James & Gregg, 2004; O'Leary et al., 2013; Ottaviani et al., 2006, 2017). However, computation of the HP score suggests that the difference in the hemodynamic profile between groups, or indeed between conditions, should be testable, rather than separating the groups to characterize the profile exhibited separately. Much of the past research has not done this almost exclusively testing the difference from zero of the HP score for each group (or experimental condition) separately. Indeed, in the current study analyses testing statistical differences between the two groups on HP directly identified no differences. However, it is clear from the use of the HP/CD model that the profiles were different. As identified by an anonymous reviewer of this article, when relative differences are the focus of research (in this case, smokers vs. non‐smokers), it seems inevitable to show that groups actually differ. When statistical tests tell us that smokers and non‐smokers do not differ in HP, but one group shows a mixed and the other group a myocardial response pattern (study 1) or one group shows a stronger myocardial response pattern than the other group (study 2), but neither of the differences is statistically significant, is this enough to conclude health‐related differences? Future research needs to adopt this core question in the use of the HP/CD model, reporting both the profile of the reaction exhibited as well as whether differences existed in the HP score. To date, research using the HP/CD model has rarely reported the latter.

The study is limited by the cross‐sectional design, with no attempt to experimentally manipulate smoking prior to the laboratory session. While all participants were asked to refrain from smoking (as well as other behaviors that may impact cardiovascular reactivity), we did not objectively measure compliance with these restrictions. Rather, we relied on self‐report to enforce exclusion/inclusion criteria. However, in Study 1, the nicotine dependency measure confirmed the self‐identified status of the smokers and social smokers in terms of nicotine dependency, suggesting at least some degree of objective verification of the self‐report of this measure. On the contrary, the findings are enhanced by the recruitment of two, independent young adult samples to assess the association of smoking status on hemodynamic profile. In addition, the consistent direction of the findings is important to note, with both samples showing no differences in cardiovascular reactivity on SBP, DBP, HR, CO, or TPR in reaction to the stressor dependent on smoking status. However, on examination of the underlying hemodynamic profile, only those who had never smoked showed the expected myocardial response to the stressor. Smokers, either past or current, did not show this expected myocardial response.

A further limitation is that analyses were underpowered to detect differences between groups on the HP score, although different profiles were identified where the HP was tested for differences from zero, as is customary in past research employing the HP/CD model of blood pressure regulation (e.g., James et al., 2012; Ottaviani et al., 2006, 2017). Future research needs to confirm the findings reported in this article, using studies specifically designed to test the impact of smoking behavior on the hemodynamic profile of the stress response in young adults. By employing more standard versions of smoking assessment (Heatherton et al., 1991), future research can seek to establish if the relationship between smoking and hemodynamic profile is characterized by a dose–response relationship. The present research was unable to establish if the degree of smoking addiction, or indeed past length of addiction, was associated with a different degree of a mixed hemodynamic response. In addition, it would be interesting to note if the blunted cardiovascular reactions to stress noted in middle‐aged samples previously reported (e.g., Ginty et al., 2014; Phillips et al., 2009; Sheffield et al., 1997) are characterized by mixed, or even, vascular patterns of stress responding.

The present study identified that, even in young adult samples, we can see evidence that smoking impacts on the reaction profile exhibited in response to active stress and is associated with a disruption of the expected myocardial response. This is important in that, while smoking may not impact on pressor or HR levels in young samples in the laboratory, it is associated with altered hemodynamic profiles in response to the stress. This identifies that smoking may not only be related to the development of heart disease through its direct effects on the cardiovascular system, but also through its impact on stress reactivity. That is, smoking status is associated with a more damaging hemodynamic profile to active stress, which, repeated over multiple stress exposures across a lifetime, may increase the risk of cardiovascular disease development through the psychosomatic pathway of stress reactivity. This is the first study to point toward the role of smoking as being associated with differing stress reaction profiles in the absence of differing blood pressure levels or responses to stress. However, how or why this is the case, is not clear. Perhaps the motivation dysregulation thought to be associated with blunted cardiovascular reactions to stress precedes the uptake of smoking; alternatively, perhaps as smoking damages the cardiovascular system, this, in turn, influences the ability of the system to react to stress appropriately. Certainly, this study identifies that whatever occurs, occurs early in the process as we see effects in these two young, healthy samples, free from disease.

## AUTHOR CONTRIBUTIONS

**Siobhán Howard:** Conceptualization; data curation; formal analysis; investigation; methodology; project administration; supervision; validation; writing – original draft; writing – review and editing. **Tracey M. Keogh:** Conceptualization; data curation; formal analysis; investigation; project administration; writing – review and editing. **Brian M. Hughes:** Conceptualization; formal analysis; investigation; supervision; visualization; writing – review and editing. **Stephen Gallagher:** Data curation; supervision; writing – review and editing.

## Supporting information

Figure S1
