# Supplementary figures and images for: Smokers show an altered hemodynamic profile to active stress: Evidence of a dysregulated stress response in young adults
Source: Psychophysiology. 2022 May 2;59(10):e14081. doi: 10.1111/psyp.14081 (PMC9541945; doi:10.1111/psyp.14081)

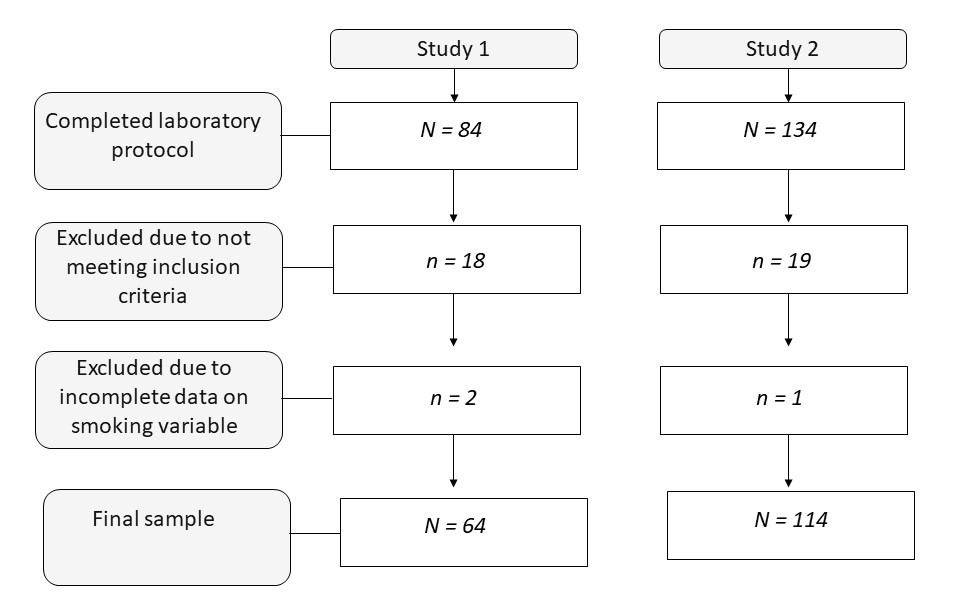

Supplement: Supplementary file 1 — Figure S1 [file PSYP-59-e14081-s001.png]
